# ABO Incompatible Deceased Donor Liver Transplants in Infants (Age < 1 Year): A Single‐Center Experience

**DOI:** 10.1111/petr.70380

**Published:** 2026-06-21

**Authors:** Heli Bhatt, Sarah Kizilbash, Claudia Cohn, Oyedele Adeyi, Daniel Waller, David M. Vock, Gwenyth Fischer, Joseph Resch, Andrew Adams, Srinath Chinnakotla

**Affiliations:** ^1^ Department of Pediatrics University of Minnesota Medical School Minneapolis Minnesota USA; ^2^ Department of Laboratory Medicine and Pathology University of Minnesota Medical School Minneapolis Minnesota USA; ^3^ Division of Biostatistics and Health Data Science School of Public Health Minneapolis Minnesota USA; ^4^ Department of Surgery University of Minnesota Medical School Minneapolis Minnesota USA

**Keywords:** allocation, liver transplant, outcomes, pediatric

## Abstract

**Background:**

The liver transplant waitlist mortality for infants (age < 1 year) is currently the highest, listed at 20 per 100 years of waiting time. ABO incompatible (ABOi) liver‐transplantation is one of the strategies to increase transplantation rate in infants but utilized only in 5.6% of infants.

**Methods:**

At our center, all children < 1 year are listed for ABOi liver transplants. If the anti‐ABO titers are less than 1:8, no plasmapheresis was used. Thymoglobulin is used for induction. We analyzed the transplant outcomes of patients that received ABOi livers compared to ABOc livers.

**Results:**

Sixteen infants, who received ABOi liver‐transplants are compared to 56 ABO‐compatible (ABOc) infant liver transplants. ABOi group utilized AB or B blood type livers 81%, compared to none in the ABOc group. The 1‐, 5‐, and 10‐year survival in the ABOi group remains at 81% and is comparable to survival in the ABOc group. None of the ABOi patients developed antibody‐mediated rejection or intrahepatic biliary strictures. None of the ABOi patients had hepatic artery thrombosis, compared with 4 (7.1%) in the ABOc group (*p* = 0.64). There is no difference in infectious complications. The median wait times for patients < 1 year for blood types A, B, AB, and were 96, 109, 82 days and 126 respectively. The patients listed to accept ABOi livers had a shorter waiting time (*p* = < 0.001).

**Conclusions:**

This study shows that ABOi liver transplants can be safely performed in children less than1 year of age without plasmapheresis, with excellent long‐term outcomes.

## Graphical abstract

The liver transplant waitlist mortality for infants (age < 1 year) is currently the highest among all ages. ABO incompatible (ABOi) liver‐transplantation is one of the strategies to increase transplantation rate in infants and can be safely performed in children less than 1 year of age without plasmapheresis, with excellent long‐term outcomes.

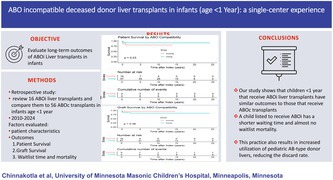

**Abbreviations**
ABO c: ABO blood type compatible liver transplant
ABOi: ABO blood type incompatible liver transplant
FFP: fresh frozen plasma
PELD: pediatric end‐stage liver disease
RBC: red blood cells
SRTR: Scientific Registry for Transplant Recipients
UNOS: United Network for Organ Sharing

## 1 Introduction

Liver transplantation for infants has excellent short‐term and long‐term outcomes. The improved outcomes are attributed to advances in medical and surgical management, particularly immunosuppression and treatment of infectious complications [1]. However, there continues to be a shortage of livers for children and particularly for infants. The waitlist mortality for children < 1 year is currently at its highest, listed at 20 per 100 years of waiting time in the United States [1]. ABO‐incompatible (ABOi) liver transplantation is one of the strategies to increase the transplantation rate in infants [2, 3].

Historically, ABOi liver transplants have been reported to have inferior patient and graft survival [4]. However, several recent reports from the United States, Europe, and Asia have confirmed that the results of ABOi liver transplants are more comparable to ABO‐compatible (ABOc) liver transplants in children than adults [2, 3, 5, 6, 7, 8, 9, 10, 11]. Despite these reports, ABOi continues to be underutilized in the United States, constituting only 4.2% of all liver transplants in infants [12].

According to the current United Network for Organ Sharing (UNOS) policy, when a patient aged 12 months or less attains a natural Pediatric End‐Stage Liver (PELD) score of 30 due to the severity of liver disease, the child can be listed to accept all blood types. At our center, we have been performing ABOi liver transplants in infants in urgent situations in the absence of suitable ABO‐identical or ABOc grafts (including technical variants and living donor grafts). The goals of this study are twofold: first we compare the transplant outcomes of patients that received ABOi livers to those of patients who received ABOc livers; second, we analyze the waiting time nationally for patients of various blood types and the discard rate of AB and B livers in the USA.

## 2 Material and Methods

### 2.1 Study Population and Data Collection

IRB approval was obtained prior to the study. The following data are collected from review of a prospectively maintained database at our institution: age at transplant; indication for transplant; PELD score at transplant; ABO type of donor and recipient; transplant‐related data, which include the donor details, type of graft, and transplant operation details; postoperative complications, including surgical re‐explorations; and long‐term outcome. Data were collected on infant ABOi and ABOc transplants that took place from January 2010 through April 2024.

### 2.2 ABOi Protocols at the University of Minnesota

#### 2.2.1 General Policy

At our center, all children < 1 year are listed for ABOi liver transplants in the absence of a potential living donor. The appropriate antibody titer (Table 1) is determined at the time of listing. When an organ offer is accepted, the titer is repeated. If this IgG or IgM titer is greater than 1:8, a temporary dialysis line is urgently placed to allow the recipient to receive plasmapheresis prior to transplant. A 1.5‐volume exchange is performed immediately prior to transplantation surgery. The antibody IgG and IgM titers are rechecked daily for 2 weeks after plasmapheresis is completed (Table 1).

**TABLE 1:** Guideline for antibody titer monitoring prior to ABO incompatible transplant.

Recipient	Antibody titer
A	Anti‐B
B	Anti‐A
O	Anti‐A & Anti‐B

#### 2.2.2 Transfusion Policy Prior to ABOi Transplant

If blood products are required while the patient is on the waiting list, then products are selected to minimize the exposure to isohemagglutinins (Table 2).

**TABLE 2:** Transfusion policy guideline for blood products prior to ABO incompatible transplant.

Donor to recipient		RBC	FFP & platelets	Anti‐Ab titer
A	O	O	A	A
B	O	O	B	B
AB	O	O	AB	A & B
A	B	B	A	A
AB	B	B	AB	A
B	A	A	B	B
AB	A	A	AB	B

Abbreviation: FFP, fresh‐frozen plasma.

#### 2.2.3 Transfusion Policy After Transplant

Following transplant, it is assumed that the endothelial cells in the liver will be replaced, and the donor liver sinusoids will be lined with the patient's native ABO antigens. However, the blood bank continues to provide components that are compatible with donor ABO type for the lifetime of the patient.

### 2.3 Post Transplant Isohemagglutinin Titers and Plasmapheresis

The IgG and IgM isohemagglutinin titer is performed with serial dilutions of the patient's plasma mixed with a 0.8% solution of the appropriate red cells daily for 2 weeks following the transplant. The mixture is applied to gel cards (Quidel Ortho Corp, San Diego, CA) and centrifuged. The titer is read as the highest dilution showing red blood cell (RBC) agglutination within the gel matrix. If the titer was noted to be > 1:8, 1.5 volume plasmapheresis is instituted. We stop checking titers after 2 weeks.

### 2.4 Transfusion Before ABOi Transplant

#### 2.4.1 Platelets & FFP

Platelets and fresh‐frozen plasma (FFP) were matched to the blood type of the donor, if known, and taken from the AB blood group if unknown (Table 2). AB platelets and AB FFP are the ideal choice for a blood type O patient who may receive an incompatible transplant before the donor blood type is known. However, AB blood type products are often in short supply. Since the blood group A antigen is the most potent antigen, blood‐type O recipients received blood‐type A platelets and FFP if group AB was not available (see Table 2).

#### 2.4.2 Red Blood Cells

Red blood cells (RBCs) were matched to the blood type of the recipient and washed to remove plasma proteins, including antibodies against RBC antigens.

### 2.5 Immunosuppression

#### 2.5.1 Induction

All ABOi patients received Thymoglobulin induction. Solumedrol 10 mg/kg was given in the operating room following the reperfusion of the newly transplanted liver. Thymoglobulin total dose was capped at 5 mg/kg body weight. If plasmapheresis was required, then Thymoglobulin was administered after plasmapheresis. Rituximab, Eculizumab, and intravenous immunoglobulin were not utilized as part of the protocol.

#### 2.5.2 Maintenance Immunosuppression

Tacrolimus and mycophenolate mofetil (450 mg/m^2^) were initiated postoperatively (Table 3). Steroids were weaned over a 1‐month period (Table 3).

**TABLE 3:** Immunosuppression protocol for ABOi livers.

Time	Tacrolimus goal ng/dL	Mycophenolate mofetil	Prednisone
0–3 months	10–12	Yes 450 mg/M^2^	Wean off at 1 month
3–6 months	8–10	Yes 450 mg/M^2^	
6–12 months	6–8	Yes wean off in the 6th month	
1–2 years	4–6		
2–5 years	3–5		
5 years onwards	1–3		

*Note:* All patients receive Thymoglobulin total dose 5 mg/Kg.

#### 2.5.3 Post‐Transplant Monitoring

Following transplant surgery, the antibody titer was measured daily for 2 weeks. Plasmapheresis was performed on a daily basis if the antibody titer was greater than 1:8. Liver tests were monitored daily for 2 weeks, after which they were monitored twice weekly and then weekly for 3 months. Patients received a protocol liver biopsy at 1 year with C4d staining, or earlier for cause.

### 2.6 Statistical Methods

Patient characteristics were summarized and compared between ABO‐compatible and ABO‐incompatible groups, calculating the mean (standard deviation) for continuous variables and the median (percentage) for categorical variables. Statistical differences between continuous variables were calculated with *t*‐tests; for categorical variables, chi‐square tests. Graft and patient survival curves were estimated using the Kaplan–Meier method, and the statistical difference between compatible and incompatible survival was estimated with a log‐rank test.

We used data from the SRTR to compare the distribution across the United States of the time to transplant between infants willing to accept an ABOi liver and those who were not. In this analysis, we included all candidates listed at less than 1 year of age during the study period (January 1, 2010–March 31, 2024). We estimated the number of days until the cumulative incidence of transplant was 30%, 50%, and 70% by blood group and willingness to accept an ABOi liver using Aalen‐Johansen estimators where death on the waiting list/removal for deterioration and removal for improvement were treated as competing events. We compared the cumulative incidence of transplant between groups using Gray's test with rho = 0.

## 3 Results

Of the 19 pediatric patients that received ABOi liver transplants at our center between January 2010–April 2024, 16 were < 1 year of age and formed the study group. They were compared to 56 ABOc liver transplants in children < 1 year performed at our center during the same time frame. The median age of the ABOi group was 6.7 ± 2.2 months, compared with 8.0 ± 2.7 months for the ABOc group (Table 3). The commonest indication for transplantation was biliary atresia. The waiting time in the ABOi group was 67 days, compared with 54 days in the ABOc group. AB or B blood type livers were used in 81% of the ABOi group (13 livers; Table 4), whereas AB and B were never used in the ABOc group. The commonest graft type was a whole liver in both groups: 32% in the ABOc group and 25% in the ABOi group received partial grafts. Although spread over a 14‐year period, all ABOi transplants except one were performed by a single surgeon (SC) and cared for by the same Pediatric intensive care and hepatology team, with minimal change in protocols.

**TABLE 4:** Pre‐ and post‐transplant characteristics.

Variable	Characteristic	ABOi	ABOc	*p*
Number of patients		16	57	
Age at transplant, mean ± SD, months		6.7 (2.2)	7.9 (2.6)	0.094
Primary cause of disease, *n* (%)	Biliary Atresia	11 (68.8)	35 (61.4)	0.70
	Alagilles	0 (0)	1 (1.8)	
	Alpha 1 Antitrypsin Deficiency	0 (0)	2 (3.5)	
	Familial Cholestasis	0 (0)	2 (3.5)	
	Metabolic Liver Disease	2 (12.5)	7 (12.3)	
	Neonatal Cholestasis	0 (0)	1 (1.8)	
	Neonatal Hepatitis	0 (0)	1 (1.8)	
	Hemangioendothelioma	0 (0)	1 (1.8)	
	Hepatoblastoma	1 (6.3)	1 (1.8)	
	TPN‐Induced Liver Disease	0 (0)	4 (7.0)	
	Other	1 (6.3)	2 (3.5)	
	Unknown	1 (6.3)	0 (0)	
Recipient blood type, *n* (%)	A	8 (50.0)	24 (42.1)	0.47
	AB	0 (0)	3 (5.3)	
	B	0 (0)	5 (8.8)	
	O	8 (50.0)	25 (43.9)	
Donor blood type, *n* (%)	A	1 (6.2)	13 (22.8)	< 0.001
	A1	2 (12.5)	7 (12.3)	
	A1B	3 (18.8)	0 (0)	
	A2	0 (0)	1 (1.8)	
	A2B	2 (12.5)	0 (0)	
	AB	4 (25.0)	0 (0)	
	B	4 (25.0)	0 (0)	
	O	0 (0)	36 (63.2)	
Portion of liver used, *n* (%)	Partial	3 (18.8)	9 (15.8)	0.61
	Split	1 (6.2)	9 (15.8)	
	Whole	12 (75.0)	39 (68.4)	
Length of hospital stay post‐transplant, mean ± SD, days		21.2 (17.0)	28.8 (25.0)	0.26
Length of hospital stay, total, mean ± SD, days		28.4 (16.8)	43.2 (55.1)	0.29
Time in ICU, mean ± SD, days		11.6 (12.6)	15.7 (18.0)	0.42
Count of rehospitalizations 1st year after transplant, mean ± SD		4.8 (4.3)	5.5 (3.9)	0.62
Waitlist time, mean ± SD		66.8 (44.2)	53.9 (51.0)	0.43
PELD score, mean ± SD		19.3 (8.6)	15.7 (11.5)	0.27

Abbreviations: ABOc, ABO compatible; ABOi, ABO incompatible; ICU, intensive care unit; PELD, pediatric end‐stage liver disease.

### 3.1 Plasmapheresis

Only one patient who had a pre‐transplant IgG and IgM antibody titer of > 1:32 in the ABOi group received pretransplant plasmapheresis and, after 1.5 volume exchange, this patient maintained a titer of less than 1:8 in the post‐transplant period. None of the 16 patients in the ABOi group needed post‐transplant plasmapheresis, and all continued to maintain low antibody titers for 2 weeks after transplant.

### 3.2 Length of Hospital Stay, Rejections and Complications

The ICU stays, total hospital stays, and rehospitalizations within the first year were similar between the groups (Table 4).

#### 3.2.1 Antibody‐Mediated Rejections

All 16 patients in the ABOi group had a protocol liver biopsy with C4d staining at 1 year, and none had evidence of acute antibody‐mediated rejection or cellular‐mediated rejection.

#### 3.2.2 Acute Cellular Rejection

Four patients in the ABOi group developed acute cellular rejection that was successfully treated with steroid pulses. There was no significant difference in the incidence of acute cellular rejection between the groups (Table 5).

**TABLE 5:** Post‐transplant complications (within 3 years).

Complication	ABOi (*n* = 16)	ABOc (*n* = 57)	*p*
Acute cellular rejection, *n* (%)	4 (25.0)	24 (42.1)	0.34
Time to rejection, mean ± SD, days	355 (591)	295 (454)	0.82
Antibody rejection, *n* (%)	0	0	NA
Hepatic artery thrombosis, *n* (%)	0	4 (7.0)	0.64
Portal vein stenosis, *n* (%)	0	0	NA
Portal vein thrombosis, *n* (%)	2 (12.5)	9 (15.8)	0.99
Biliary Stricture, *n* (%)	0	13 (22.8)	0.082
Time to biliary stricture, mean ± SD, days	NA	110 (194)	NA
CMV infection, *n* (%)	3 (18.8)	11 (19.3)	0.99
PTLD, *n* (%)	1 (6.2)	5 (8.8)	0.99
Bacterial infections, *n* (%)	12 (75.0)	45 (78.9)	0.99
Respiratory, *n* (%)	5 (31.2)	10 (17.5)	0.40
Bloodstream, *n* (%)	1 (6.3)	12 (21.1)	0.32
Gastrointestinal, *n* (%)	3 (18.8)	4 (7.0)	0.35
ENT, *n* (%)	1 (6.3)	3 (5.3)	0.99
Urogenital, *n* (%)	1 (6.3)	0	0.49
Skin/sub cut, *n* (%)	1 (6.3)	1 (1.8)	0.91
Liver/biliary, *n* (%)	0	14 (24.6)	0.065
Unknown site, *n* (%)	0	3 (5.3)	0.82

Abbreviations: ABOc, ABO compatible; ABOi, ABO incompatible; CMV, cytomegalovirus; ENT, ear, nose, throat; NA, not applicable; PTLD, post transplant lymphoproliferative disease.

#### 3.2.3 Post‐Transplant Complications

##### 3.2.3.1 Surgical Complications

Complications occurring within 3 years after the transplant are shown in Table 5. Biliary complications were higher in the ABOc group (Table 5), but not significantly so (*p* = 0.082). None of the ABOi patients developed intrahepatic biliary strictures. None of the ABOi patients had hepatic artery thrombosis, compared with 4 (7.1%) in the ABOc group (*p* = 0.64). There was no difference in the portal vein complications between the groups. There was no difference in complications between those receiving partial livers versus whole liver graft.

##### 3.2.3.2 Infectious Complications

Bacterial respiratory infections were the most common infections in both the groups, followed by bloodstream infections, but neither was significantly different between the groups (Table 5).

Cytomegalovirus infection occurred in 3 ABOi patients (19%) and 11 ABOc patients (19.3%) (*p* = 0.99). The EBV viral loads at 3 months were not different between the groups. Post‐transplant lymphoproliferative disorder occurred in 1 patient (6%) in the ABOi group and 5 in the ABOc group (9%) (*p* = 0.99, Table 5). All the ABOi recipients received Thymoglobulin induction compared to Basiliximab in the ABOc group, and there were no differences in infectious complications between the groups.

### 3.3 Patient Survival

The 1‐year, 5‐year, and 10‐year survival in the ABOi group remained at 81% and was comparable to survival in the ABOc group (Figure 1a).

**FIGURE 1a:**
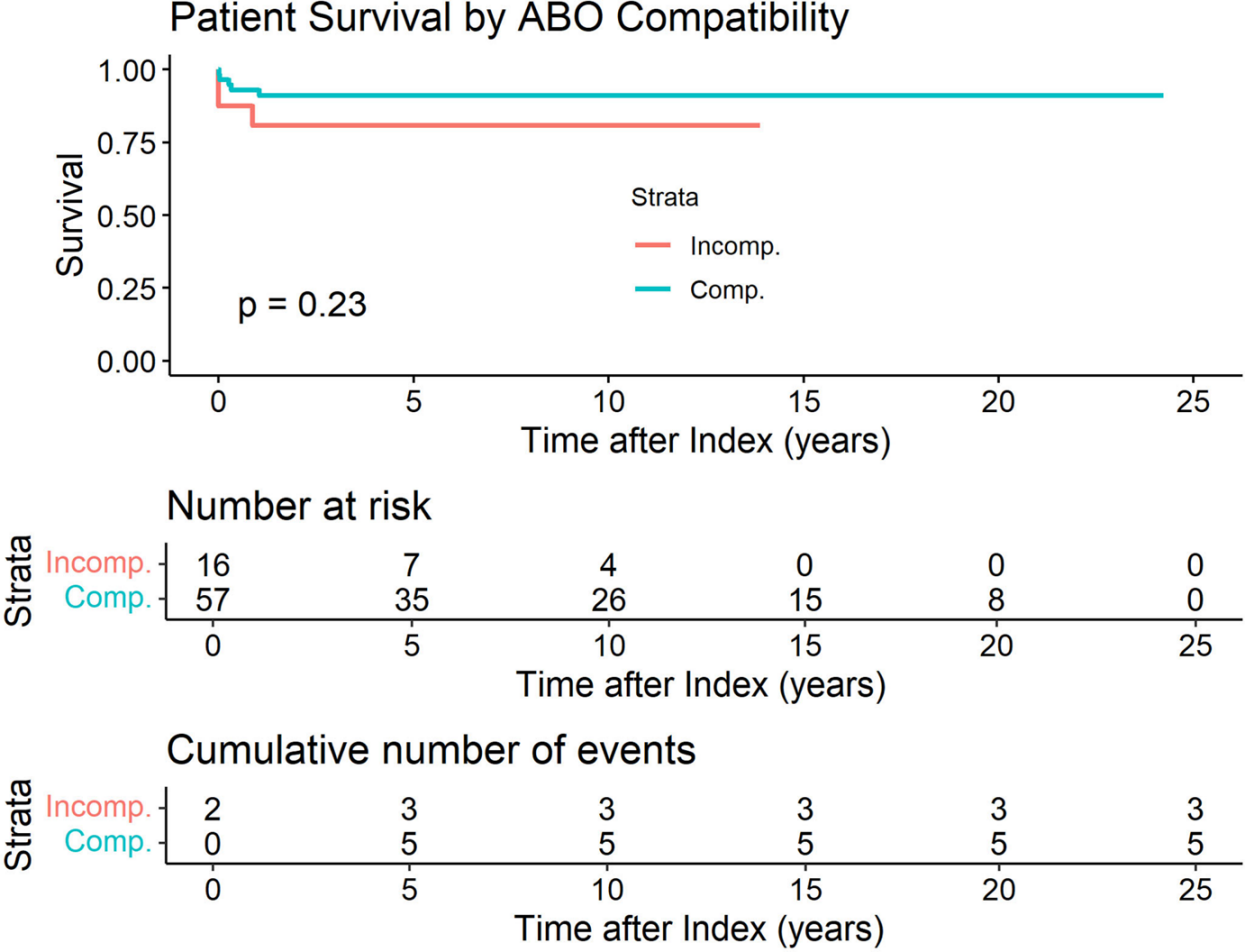
Patient survival among ABO incompatible liver transplant patients compared to ABO compatible transplants.

The causes of death in the ABOi group are described below:

Patient 1. A 2‐month‐old male patient was listed as Status 1A for acute liver failure. The patient bled from a pretransplant liver biopsy and was on 3 pressors prior to transplant. A CT scan before the transplant showed viable bowel, but at surgery the entire small bowel was noted to be gangrenous. The bowel was resected, and an attempt was made to perform the transplant, but after portal vein anastomosis, the patient coded and could not be revived.

Patient 2. A 4‐month‐old male patient was listed as Status 1A with acute liver failure and was transplanted with a left lobe reduced graft. After releasing the portal vein clamps, the patient experienced cardiac arrest and could not be revived.

Patient 3. A 9‐month‐old patient with biliary atresia listed with PELD exception score 35 was transplanted with a whole liver. The transplant was uneventful, and the patient was discharged with excellent liver graft function. The patient was readmitted 9 months later with respiratory failure and died on postoperative day 313 due to acute respiratory distress syndrome of unknown etiology. The infectious workup was negative.

In patient #1 and patient #2, the deaths were unrelated to the ABO specific complications. We excluded these two cases and re‐calculated the patient survival, which is presented in Figure 1b, to remove the overestimation of ABOi related risk of mortality.

**FIGURE 1b:**
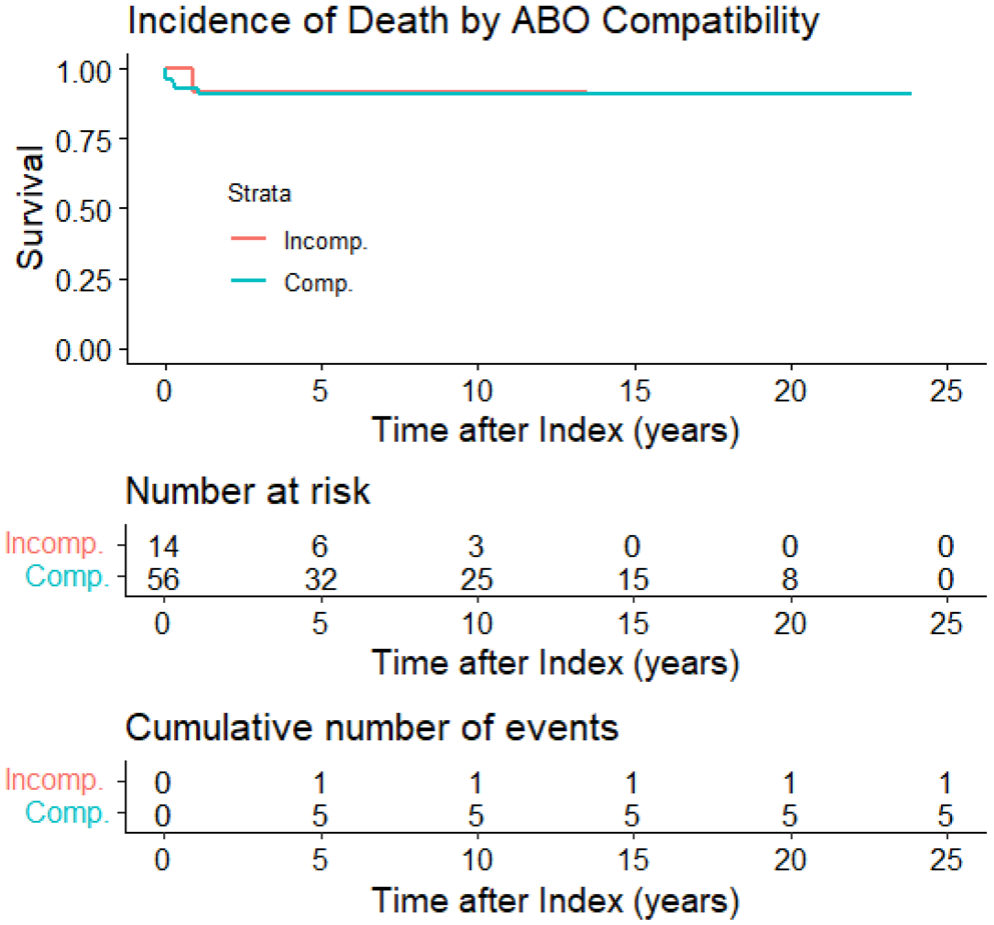
Patient survival by ABO Compatibility excluding the 2 intra operative deaths that occurred prior to complete implantation of the liver graft and unrelated to the ABOi risk.

There were 4 deaths in the ABOc group from vascular thrombosis (2), primary graft failure (1), and pulmonary hemorrhage (1).

### 3.4 Graft Survival

**FIGURE 2:**
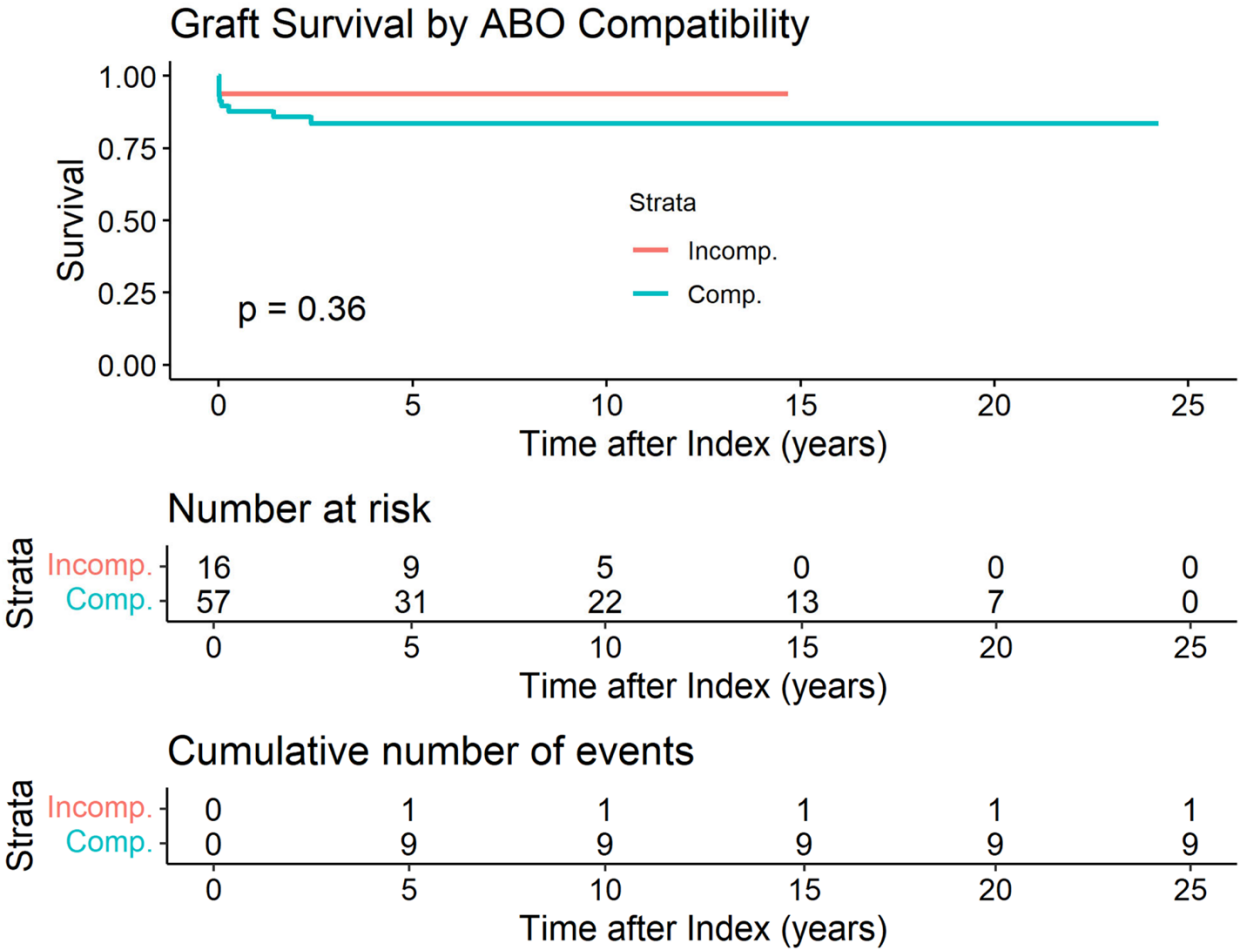
Graft survival among ABO incompatible liver transplant patients compared to ABO compatible transplants.

One patient had graft loss due to inadequate portal inflow resulting from portal vein thrombosis on postoperative day 2. This patient was successfully re‐transplanted with an ABOc graft using the recipient renal vein as inflow. The 1‐year, 5‐year, and 10‐year graft survival in the ABOi group remained at 94% and was comparable to that in the ABOc group (Figure 2).

### 3.5 Time on National Waitlist and Waste of AB Livers

The median wait times for patients < 1 year for blood types A, B, AB, and were 96 days, 109 days, 82 days, and 126 days respectively. The patients listed to accept ABOi livers had a shorter waiting time (Table 6).

**TABLE 6:** Time (days) until a cumulative incidence of transplanted reached 50% among those listed at < 1 year age in the United States between January 2010 and April 2024 by blood type. Aalen‐Johansen estimators were used treating death on the waiting list/removal for deterioration and removal for improvement as competing events. Numbers in parentheses are the time to cumulative incidence of 30% and 70%. *p*‐value is from Gray's test comparing the cumulative incidence of transplant.

Blood type	Listed to accept ABOi liver (*n* = 583)	NOT listed to ABOi liver (*n* = 2755)	*p*
A	50 (15–119)	107 (46–326)	< 0.001
B	51 (20–155)	120 (54–350)	0.001
AB	14 (10–95)	85 (41–148)	0.64
O	111 (22–126)	155 (70–552)	< 0.001

The non utilization of blood type AB livers and B livers among donors less < 18 years in USA during the same time was 5.93% and 5.9% respectively.

## 4 Discussion

Although liver transplantation has enjoyed tremendous success in the last decade, children less than 1 year old continue to have the highest mortality on the waitlist [1]. Various responses have been developed, which include split‐liver transplantation, living donation, and wider acceptance of so‐called marginal grafts such as grafts from donation after cardiac death. However, the use of ABOi donors seems to have lagged behind and constitutes only 5.6% of all pediatric liver transplants performed in the United States [12]. The initial results with ABOi were unsatisfactory, with high rates of rejection, hepatic artery thrombosis, biliary complications, sepsis, and other causes of inpatient graft loss. It has been previously noted, however, that the results of ABOi liver transplants are better in children than in adults and better still in the children < 1 year old [2, 4, 13]. The use of ABOi livers continues to be limited to the extreme circumstances of patients with urgent need of liver transplantation. The current UNOS policy limits infants to be listed for all blood types only if their listing PELD score is 30 [14].

Although the numbers are small, our study shows that the results of ABOi liver transplants are comparable to those of an ABOc liver transplant, with minimal use of plasmapheresis and no associated antibody‐mediated rejection. The practice of listing patients for all blood types decreases the waiting list time and potentially could eliminate waitlist mortality in children < 1 year of age. The reason for limiting the use of ABOi livers to children < 1 year was twofold (i) the isohemagglutinin production typically begins around 6 months of age and complement activation is attenuated in infants, thus this age group has relative immunologic tolerance and (ii) < 1 year children have the highest mortality on the liver waitlist, thus have the most need [1, 13, 15, 16]. We anticipate that this should set a benchmark for the use this source of liver grafts for transplantation in children < 1 year old. Valentino et al. recently reported similar findings in a series of 25 pediatric patients, where the time on waitlist for ABOi recipients was shorter (median 11 days compared to 113 days for ABOi) with similar results post transplants. The majority of the liver grafts utilized in our cohort were whole livers. Our center is centrally located in US and tends to receive more organ offers and the OPO's in US are very aggressive in pursuing pediatric organ donation thus the higher use of whole liver grafts compared to other studies. The organ acceptance ratio at our center also is 1.6 [1.32–1.89] 95% credible interval higher than expected nationally [17].

Markiewicz‐Kijewska et al. reported on 142 patients with ABOi transplant from 8 European transplant centers, which included 78 patients who were 1 year or younger [18]. They found that children ≤ 1 year had overall survival of 77%, compared with 70% in children > 1 year of age, which was not statistically significant [18]. Older children had a higher incidence of intrahepatic biliary strictures and re‐transplantation rate than younger children. These findings are similar to the experience from Egawa et al., who reported that 5‐year patient survival for patients < 1 year old and > 16 years old were 77% and 22%, respectively [16]. The reasons for the better outcomes in children < 1 year are not clear, though may be related to the lower anti‐ABO titers noted in children aged < 1 year, as we saw in our cohort [16]. The immaturity of the immune complement system in children < 1 year may also be helpful [16].

Two of the deaths in our cohort occurred in patients listed with status 1A. Lemoine et al. reported their experience of 17 patients that received 18 ABOi transplants over a 23‐year period. Urgent Status 1A patients had an inferior patient survival (63% 1‐year survival) than patients listed with a PELD score (100%) [14]. The authors recommended that the indications should be liberalized for patients with high PELD scores to prevent death and deterioration on the waiting list [14]. The Markiewicz‐Kijewska et al. European multicenter report [18] also noted that acute/urgent liver transplants tended to have inferior survival than elective liver transplants. Patients with vascular complications were less likely to survive.

Gautam et al. reported on 20 children that received ABOi liver transplants and found that ABOi recipients had a higher incidence of bile leaks and prolonged hospital stay. However, other complications like acute cellular rejection, sepsis, and vascular complications had similar rates as those seen after ABOc liver transplants [6]. Unlike our cohort, Gautam et al. used an intense desensitization protocol consisting of rituximab (375 mg/m^2^) and plasmapheresis if the isohemagglutinin titer was greater than 1:16 [6]. Like our cohort, none of their patients were greater than 1 year of age. They also had a mortality of 21% [6]. Eculizumab is a monoclonal antibody that binds to complement protein C5, preventing its cleavage into C5a and C5b and thereby inhibiting the formation of the terminal complement complex C5b‐9, also known as the membrane attack complex [19]. Eculizumab has been successfully utilized for de‐sensitization option before an ABOi transplantation without plasmapheresis and is beneficial for patients who are listed for Status 1A and are hemodynamically unstable, coagulopathic, and unstable for plasmapheresis [19]. Egawa et al. reported that patients 1–8 years with high pretransplant anti‐ABO titers remain at considerable risk of fatal outcome because of development of intrahepatic biliary strictures or hepatic necrosis and suggest restricting ABOi in patients of these ages [2, 5].

### 4.1 Immunosuppression and Rejection

The use of pediatric desensitization protocols for ABOi in children is variable among transplant centers and includes splenectomy, plasmapheresis, immunoadsorption, high‐dose intravenous immunoglobulin, and anti‐CD20 monoclonal antibodies [18, 20]. In the European study, 8% of all recipients received some form of desensitization [18]. In reports from Asia, rituximab was more commonly used [6]. In the United States, plasmapheresis is routinely used in several centers. Data suggest a direct relationship between antibody‐mediated rejection and pre–liver transplant antibody titers of greater than 1:16 [2, 5]. In the current study, although we used a threshold of 1:8 for performing a pretransplant plasmapheresis, only one of our patients required it, and none required plasmapheresis in the post‐transplant period [11]. We did not see any evidence of acute antibody‐mediated rejection in our cohort. An anti‐donor antibody titer of 1:16 has been reported in the Japanese literature to be associated with a higher risk of antibody‐mediated rejection after ABOi adult living‐donor liver transplants, and this number was used by the Japanese group to initiate pretransplant plasmapheresis [5]. The induction regimen used in our protocol included Thymoglobulin, and we did not utilize Rituximab, and we were able to achieve excellent long‐term outcomes. Thymoglobulin is a polyclonal antibody and potentially exerts immunomodulatory effects beyond initial T cell depletion; it possibly mediates immunomodulation and graft tolerance by functionally inactivating cell surface receptors involved in antigen recognition, leukocyte trafficking, and leukocyte endothelium adhesion [21]. Other programs have successfully utilized basiliximab with excellent outcomes [7]. The role of basiliximab needs to be investigated further in future studies.

### 4.2 Technical Complications

Children receiving ABOi grafts are at increased risk of developing vascular complications [4]. Early humoral rejection resulting in massive intrahepatic microcirculation endothelial damage and thrombosis may cause hemorrhagic graft necrosis and loss within a few days [5, 20]. Fortunately, this is not commonly seen in pediatric patients but may contribute to an increased incidence of hepatic artery thrombosis, which has been reported in other studies to be as high as 24% [4]. In our series, there were no differences in technical complications between ABOi and ABOc groups. Fewer than 10% developed hepatic artery thrombosis. Patients that develop vascular complications have a higher risk of graft loss and patient death [2]. At our center, we use heparin and aspirin in this subgroup of patients to prevent vascular thrombosis and have been able to prevent vascular complications. Another complication that may occur within the first 3 months after ABOi is damage to intrahepatic bile ducts and development of multiple intrahepatic biliary stenoses [2]. This is caused by immunological insult by humoral reaction to the donor blood antigens, which are present on the epithelium of the bile ducts for 3–6 months after liver transplantation. In our series, maintaining the antibody titers < 1:8 perhaps avoided this complication.

### 4.3 Long‐Term Follow‐Up

In our cohort, the graft and patient losses occurred early on. Once the patient survived past 1 year, they did not have any evidence of antibody‐mediated rejection nor any other complications. This mimics the reports of infant ABOi heart transplantation, where donor‐specific tolerance was achieved by elimination of donor‐reactive B lymphocytes due to intentional exposure to nonself A and B antigens [22]. Another plausible explanation is “immunological accommodation”; despite the presence of anti‐A/B antibodies against those antigens in the blood of the recipient, no antigen–antibody reaction (i.e., acute antibody‐mediated reaction) occurs [5, 16]. This donor‐specific hyporesponsiveness has been shown to remain in pediatric liver transplant recipients [23], and the long‐term persistence of red blood cell antigens may contribute to donor‐specific hyporesponsiveness. This could possibly explain the excellent long‐term graft outcome in our patients past the 3‐month period [5].

### 4.4 Limitations of the Study

This was a retrospective study, and the information analyzed was limited to the information documented in the charts. The numbers were too small for any subgroup analyses, and the patients were spread over a 14‐year period.

### 4.5 Implications of the Finding of Our Study

Our study shows that children < 1 year that receive ABOi liver transplants have similar outcomes to those that receive ABOc transplants. A child listed to receive ABOi has a shorter waiting time and almost no waitlist mortality. This practice also results in increased utilization of pediatric AB‐type donor livers, reducing the discard rate. These data support changing the current UNOS policy to allow listing all infants to accept all blood types, reducing waiting times and reducing waitlist mortality.

In summary, our present study shows that ABOi liver transplants can be safely performed in children less than 1 year of age by using Thymoglobulin induction without any plasmapheresis, resulting in excellent long‐term outcomes. Further larger studies are necessary to validate the results.

## Funding

This work was supported by Kristina L. Scheid.

## Conflicts of Interest

The authors declare no conflicts of interest.

## Data Availability

The data that support the findings of this study are available from the corresponding author upon reasonable request.
